# Unmet needs in cellulitis diagnosis: a qualitative interview study to understand healthcare professionals’ knowledge and experiences

**DOI:** 10.1136/bmjopen-2026-117201

**Published:** 2026-06-28

**Authors:** Elizabeth LA Cross, Katy Baple, Martin J Llewelyn, Margaret Glogowska, Gail N Hayward

**Affiliations:** 1 Centre of Infection and Antimicrobial Research (CINAMR), Brighton and Sussex Medical School, University of Brighton and University of Sussex, Brighton, UK; 2 Department of Infection, Royal Sussex County Hospital, University Hospitals Sussex NHS Foundation Trust, Worthing, England, UK; 3 Nuffield Department of Primary Care Health Sciences, University of Oxford, Oxford, UK; 4 NIHR HealthTech Research Centre in Community Healthcare, Oxford, UK

**Keywords:** Infectious diseases & infestations, INFECTIOUS DISEASES, Diagnostic microbiology, Adult dermatology, Geriatric dermatology

## Abstract

**Abstract:**

**Objective:**

To explore healthcare professionals’ (HCPs) experiences of diagnosing cellulitis, identify unmet diagnostic needs and determine the desirable characteristics of an ideal diagnostic test.

**Design:**

Qualitative study using semistructured interviews, analysed thematically.

**Setting:**

Community and secondary care services across the UK from January 2023 to September 2023.

**Participants:**

25 UK-based HCPs (eg. nurses, paramedics, pharmacists, general practitioners and hospital physicians) with recent experience of managing cellulitis. Inclusion required recent involvement in cellulitis care; no specific exclusion criteria were applied. All participants completed the interview.

**Results:**

Four major themes were identified. Theme 1 described patient and disease characteristics that make diagnosis difficult, including atypical or recurrent presentations, high-risk patients, pre-existing wounds or ulcers, darker skin tones and prior antibiotic use, which represented specific use cases in which a test could be deployed. Theme 2 highlighted clinician and patient behaviours contributing to misdiagnosis, including reliance on cellulitis as the ‘default’ diagnosis, limited dermatology/tissue viability knowledge and access, risks of not treating and patient expectations. Theme 3 examined candidate diagnostic aids (evaluated in early studies) and ideas for novel diagnostic tools, noting that current tools are insufficiently specific, while enthusiasm for new technologies was countered by concerns about usability, comfort, infection control and training needs. Theme 4 identified key characteristics of an ideal test: portable, rapid, equitable, usable across community and hospital settings and providing a probabilistic result rather than a binary outcome.

**Conclusions:**

Cellulitis remains diagnostically challenging across multiple care settings. Developing tools that can distinguish patients who genuinely require antibiotic therapy from those with non-infective inflammation would be particularly valuable. The greatest potential for impact lies in improving diagnostic specificity to reduce misdiagnosis, unnecessary antibiotic use and avoidable healthcare attendance and admission. Broad engagement of stakeholders will be essential to define acceptable performance parameters and guide novel technology development.

**STRENGTHS AND LIMITATIONS OF THIS STUDY**

- The sample included healthcare professionals from multiple care settings, enabling exploration of diagnostic needs across the patient pathway.
- Use of double coding, reflective logs and regular team discussions enhanced trustworthiness and credibility.
- Some questions required participants to comment hypothetically on potential diagnostic tools, which may limit the applicability of these responses.
- As a UK-based study, findings may not fully reflect diagnostic pathways or specialist access in other healthcare systems.

## Introduction

 Cellulitis is a bacterial skin infection that classically presents with an acute onset of pain, warmth, swelling and spreading colour change in the affected area.^1^ Any area of skin may be affected, but the lower limb is the site of infection in 70%–80% of patients.^2^

Cellulitis is primarily a clinical diagnosis, based on medical history and physical examination, sometimes supported by non-specific diagnostic tests such as blood inflammatory markers.^1 2^ Other skin, vascular and inflammatory disorders present in similar ways and so can be mistaken for cellulitis by healthcare professionals (HCPs).^3^ Studies conducted in the USA, UK and Canada in both inpatient and outpatient settings report a cellulitis misdiagnosis rate of ~40%.^4^

One US study has estimated that misdiagnosed cellulitis results in 50 000–130 000 avoidable hospital admissions and US$195–515 million in healthcare costs per year.^5^ Misdiagnosis also leads to unnecessary antibiotic use, fuelling antibiotic resistance, an urgent threat to human health.^6^

The lack of validated diagnostic aids has been highlighted as a key limitation in effectively managing cellulitis and its mimics.^7^ There are few emerging novel approaches to aid the diagnosis of cellulitis, and the evidence is not robust enough to support their routine use in clinical settings.^7 8^

The development of new diagnostic tests first requires understanding of the current diagnostic process and what test characteristics are desirable. Therefore, we aimed to explore HCPs’ experiences and unmet needs in cellulitis diagnosis and what they felt would constitute an ideal diagnostic test.

## Methods

We used a qualitative methodology, which is appropriate for exploring and understanding decisions, actions and processes. We drew on principles of grounded theory to guide our data collection and analysis. In practice, this meant that we used early interviews to inform our approach to sampling and identifying further participants for interview, in order to capture as wide a range of experiences as possible (theoretical sampling). Our chosen method was semistructured interviewing which allows exploration of topics of interest but also allows participants to raise issues of significance to them. The data collected in the interviews were analysed thematically.

### Study participants and sampling

We interviewed HCPs (nurses, paramedics, pharmacists, General Practitioners (GPs), and doctors from acute, general, infectious diseases, microbiology and dermatology specialties) who had recent experience caring for patients with cellulitis in community and/or hospital-based settings across the UK from January 2023 to September 2023. Participants were invited to participate through advertisements on social media platforms, other web-based platforms (eg, professional websites), professional mailing lists and snowballing from professional contacts. We aimed for professional diversity of participants in our early interviews and then identified further participants to capture perspectives we considered to be of interest. We planned to conduct up to 30 interviews, but by interview 25, sufficient information had been obtained to explain the emerging themes,^9^ and the decision to stop interviewing was agreed on among members of the research team.

### Data collection and analysis

Interviews lasted up to 1 hour and were conducted either online or by telephone, according to the participant’s preference.

A topic guide was developed from the existing literature and the research team’s previous experiences in cellulitis research (see online supplemental materials). The topic guide was piloted with the initial five interviews and amended accordingly. Further adaptation occurred in the light of ongoing analysis to fully explore emerging ideas and themes. Interviews were digitally audiorecorded and transcribed for data analysis. Transcripts were checked, and data were handled using NVivo software (V.14).

Interviews were conducted by two medically qualified members of the research team. KB conducted 20 interviews and was a resident doctor in general medicine with approximately 4 years postgraduate experience and no specific expertise in cellulitis research. ELAC conducted five interviews and was a more senior resident doctor with approximately 8 years postgraduate experience, specialising in infectious diseases and undertaking a doctoral degree in the clinical evaluation of cellulitis.

ELAC read the transcripts to familiarise herself with the data and conducted line-by-line coding. KB also independently coded the first five transcripts. Data were analysed thematically using the method of constant comparison.^10^ This allowed the researchers to move between different parts of the data set in order to ascertain that issues and categories developed in one part were present elsewhere so that all the data were explored and included in the analysis. Through this iterative process, the coding framework was refined, and consensus was reached among the research team regarding the interpretation of key themes.^10^

To ensure the trustworthiness and credibility of the data analysis, we reviewed the reflective logs completed after each interview which supported the emerging themes. These findings from the study were then reviewed in research meetings to see how they resonated with the experiences of other clinicians in the research team and with the existing literature. Confirmability was established using a careful audit trail setting out the decisions taken in data collection and data analysis.^11^ We used the Standards for Reporting Qualitative Research (SRQR) reporting guideline^12^ to draft this manuscript, and the SRQR reporting checklist^13^ when editing, included in the online supplemental file 2.

### Patient and public involvement

Patients and/or the public were not involved in the design, conduct, reporting or dissemination plans of this research.

## Results

Of 25 HCPs interviewed; five participants had <10 years of experience, 10 had 10 to <20 years and nine had >20 years. Most participants (80%) had diagnosed a case of cellulitis within the preceding month, 76% had diagnosed >50 cases of cellulitis, and 96% were prescribers. Their other characteristics are outlined in table 1.

**Table 1:** Participant characteristics

Participant no.	Job title/role	Work setting	Number of cellulitis diagnoses	Time since last diagnosis
1	GP (salaried)	GP surgery	10–50	>2 months
2	GP (OOH)	GP surgery and community	>50	2 months
3	Emergency nurse practitioner	Emergency department	10–50	1–2 weeks
4	GP (locum)	GP surgery and UTC	>50	>2 months
5	Advanced district nurse specialist	Community	>50	1 month
6	Lead antimicrobial pharmacist	DGH	10–50	1–2 weeks
7	District nurse	Community	10–50	<1 week
8	Acute medicine consultant	Teaching hospital	>50	<1 week
9	GP (OOH)	Community and UTC	>50	1–2 weeks
10	Consultant antimicrobial pharmacist	Teaching hospital	>50	<1 week
11	Infectious diseases consultant	DGH	>50	1–2 weeks
12	Microbiology consultant	Teaching hospital	10–50	<1 week
13	GP (salaried)	GP surgery	>50	1 month
14	Acute medicine and cardiology consultant	DGH	>50	1–2 weeks
15	Dermatology nurse consultant	Outpatients	>50	2 months
16	Acute medicine consultant	DGH	>50	1–2 weeks
17	Emergency nurse practitioner	Community hospital minor injuries unit	10–50	1–2 weeks
18	Acute medicine consultant	DGH	>50	<1 week
19	Emergency medicine trainee	DGH	>50	>2 months
20	Advanced clinical practitioner	Teaching hospital	>50	1–2 weeks
21	Advanced nurse practitioner	Teaching hospital	>50	<1 week
22	Infectious diseases and microbiology consultant	DGH	>50	<1 week
23	Microbiology consultant	DGH	>50	<1 week
24	Dermatology nurse specialist	Outpatients	>50	1 month
25	Advanced clinical nurse practitioner	GP surgery, mainly dermatology	>50	1–2 weeks

DGH, district general hospital.; GP, General Practitioner; OOH, out of hours; UTC, urgent treatment centre.

Participants described a range of challenges and barriers to the accurate diagnosis of cellulitis and what they perceive to be the ideal characteristics of a diagnostic test, which we grouped into four themes: the first describes patient and disease characteristics that make diagnosis difficult, the second outlines how clinician and patient behaviours may contribute to misdiagnosis, the third describes HCPs’ views on candidate and novel diagnostic solutions and the fourth is concerned with ideal diagnostic test characteristics. From the first theme emerged five distinct ‘use cases’ (specific scenarios or situations) in which an accurate diagnostic test could be applied; these are described in table 2.

**Table 2:** Links between use cases and ideal test characteristics within theme 1

Theme	Use cases	Ideal test characteristics
Host and disease characteristics that make diagnosis challenging	Non-classical phenotypes	Users: less experienced HCPs
	High-risk patients Ties in with: Devolving diagnostic responsibility safely to less experienced HCPs Improving communication between patients, nursing home / non-medical staff and the primary care team	Setting: home-based, long-term care facilities, ambulance service. Users: self-administered by a trained member of the public or carer, non-medical staff, home visits by community or urgent response teams. Portable, robust, quick time to result, easy to train (eg, point-of-care/rapid/bedside test), result format can indicate whether expert/medical review is required and the timeframe for this, rechargeable, high sensitivity.
	Pre-existing wounds or ulcers	Users: district nurses, practice nurses, dermatology nurses. Portable, robust, quick time to result, rechargeable, non-contact based or protective barrier/covering, clear decontamination procedures.
	Different skin tones	If it is a visual/optic/thermal/artificial-intelligence-based technology, then it needs to be validated in different skin tones.
	Previous oral antibiotic therapy	Setting: secondary care, therefore, the time to result might not have to be as quick.

### Theme 1: host and disease characteristics that make diagnosis challenging

#### Non-classical phenotypes

Participants described the ‘classic’ presentation of cellulitis as simple to diagnose. However, they indicated that a diagnostic test would be useful for the other phenotypes of the disease, such as in less acutely unwell patients and those with pre-existing skin changes, particularly if they had recurrent presentations that could be due to inflammation rather than infection.

“the lower grade cellulitis’ where somebody’s not unwell with it are particularly challenging” (P2 GP, out of hours (OOH))

*“*So I think it can be clinically barn door when you’re looking at them but actually sometimes it can be really difficult, and then you start to think well, these patients with poor venous circulation are more likely to get cellulitis, so they’re at risk and therefore is this an infection on top of the other bit of venous insufficiency? And that’s when it’s really challenging*.”* (P8 acute medicine consultant)

“it’s the recurring cases that we’ve found particularly tricky…It’s the ones that have got underlying lower limb deficiency, where they just keep bouncing back…it all goes away with antibiotics and then you get four weeks down the line and it’s all flared up again…Yes, it’s just so tricky knowing what we do with them and whether the inflammation truly is infection*.”* (P25 advanced clinical practitioner)

#### High-risk patients

Participants said that a test would be of value for patients who they consider to be at high risk of deteriorating due to frailty and multiple comorbidities, often also the population who require home visits.

“those people who are unable to travel…are inevitably the sickest because of multiple pathology and they are often the ones that it’s most difficult to make a diagnosis on and to decide an appropriate treatment pathway…they’re the highest risk patients.” (P9 GP, OOH)

#### Pre-existing wounds or ulcers

Even participants with experience in managing patients with pre-existing wounds or ulcers found diagnosis particularly challenging in this patient population.

“So, you’ve got patient that comes in – they’ve got cellulitis – or they think they’ve got cellulitis – but also leg ulceration can look very similar to cellulitis. A leg ulcer can be very clinically red – it can be painful – it can smell – it can have pus – it can have slough – which can be…very difficult, sometimes, for people to see a big erythematous leg and think it’s infected when, actually, not – it’s just a poorly-managed leg ulcer.” (P24 dermatology specialist nurse)

#### Different skin tones

Some participants highlighted that a diagnostic test or tool might help diagnose cellulitis in people with darker skin tones, as this was considered a particular challenge.

“I think there is such a huge variation in skin tones and textures and cellulitis in a person of non-white European skin colour, appears so differently.” (P6 lead antimicrobial pharmacist)

#### Previous oral antibiotic therapy

Participants also described the clinical challenge of a patient who has already received some oral antibiotic therapy. In these situations, it is possible that oral antibiotics were unable to treat the infection adequately; however, the cellulitis diagnosis could also have been incorrect. Therefore, a diagnostic test that could help distinguish between a poor response and an incorrect diagnosis would be valuable.

“And perhaps they’ve had oral antibiotics and haven’t responded. Because we’ll often get referrals from GP’s where patients have had a trial of oral antibiotics and it’s not a clear cut case of cellulitis, but they’ve got a persistently swollen leg. And the GP wants us to consider IV antibiotics.” (P16 acute medicine consultant)

### Theme 2: clinician and patient behaviours contributing to misdiagnosis

#### Clinician behaviours

##### Cellulitis is the default diagnosis

HCPs reported that they might be more likely to label patients with cellulitis incorrectly as the steps involved in diagnosis and treatment are more straightforward than for the other differentials.

“It’s because it’s an easy diagnosis to make and an easy diagnosis to treat and it is common, so because it’s a common diagnosis you’re likely to be right a lot of the time…and the treatment is usually pretty well tolerated…and you can arrange a safety net as well in terms that you’re usually reviewing them in a few days down the line to see how they’re getting on.” (P18 acute medicine consultant)

##### Poor dermatology knowledge and specialist access

HCPs also acknowledged that a cellulitis diagnosis was more likely because of poor knowledge of other dermatological conditions coupled with a lack of timely access to a specialist opinion/care.

“I know from my experience, my dermatology isn’t up to scratch, so it is easier to think it’s a bacterial infection that could be fixed with a tablet and off you go, than thinking actually, what else could this be dermatology wise…I’ve got no idea, but I do know that I can give someone flucloxacillin…if it’s not cellulitis, having an ease of getting through to potentially tissue viability or dermatology, that’s a big barrier of what do we then do…we can take photos, we can send it off, but that’s not the same day sort of response.” (P20 advanced nurse practitioner)

##### Risks of not treating versus overtreating

Participants also described making a cellulitis diagnosis more frequently because they felt the consequences of missing the diagnosis would usually be worse than treating someone unnecessarily with antibiotics.

“Just because of the balance of risk. So yeah, even though we do want to avoid giving people antibiotics who don’t need them, it’s probably safer for that to happen than for someone who needs them to go without.” (P7 district nurse)

### Patient behaviours

#### Going against patients’ expectations for antibiotics

HCPs described patient expectations for antibiotics as a barrier to questioning the label of cellulitis (given either by a previous HCP or themselves) and whether antibiotics are the correct treatment.

“so they’re referred to us and they expect either a hospital admission or intravenous antibiotics so I think patient’s expectation of what they’re going to get is a big barrier…it’s trying to manage the patient’s expectations while you’re trying to figure out what’s the best course of treatment because just antibiotics isn’t necessarily the right course of treatment.” (P20 advanced clinical nurse practitioner based in a GP practice)

#### Challenging a pre-made diagnosis can damage trust in other HCPs

If a patient has already started treatment, clinicians can be concerned that challenging a diagnosis a patient has already been given could cause anxiety and undermine confidence. A diagnostic tool/device could be beneficial in managing that and in increasing trust.

“Yeah. I think then you could phrase it with the patient in such a way…‘I’ve got this device and this has allowed me to say fairly confidently that this is unlikely to be cellulitis.’ Then you can have that conversation, ‘It’s very difficult to diagnose cellulitis, your GP or your nurse was just trying to keep you safe, and that’s why they prescribed it and it’s not because they were wrong, but because they erred on the side of caution.’…it’s not you against the other clinician that’s looking after them, it’s like you’ve got a device that’s sort of neutral.” (P15 dermatology nurse consultant)

### Theme 3: candidate and novel diagnostic solutions

#### Candidate diagnostic aids

During the interview, the researcher briefly introduced three main categories of candidate diagnostic aids (evaluated in early studies) for cellulitis (thermal measurement technologies, scores or clinical decision support systems and point-of-care blood tests). In general, participants valued aids that were simple, rapid and non-invasive, particularly where they could provide reassurance to decision-making. However, there were concerns that all the tools lacked specificity to differentiate from other inflammatory differentials, might over-simplify complex clinical assessments or could detract from holistic clinical judgement. Positive and negative perceptions of each type of aid are presented in table 3.

**Table 3:** Perceptions of candidate diagnostic aids

Type of aid	Positives	Negatives
Thermal measurement	Non-invasive No need for bodily fluids	Perceived as insufficiently specific to rule out inflammatory mimics Consider touch-based assessment of temperature to be adequate Temperature cut-off may be too small in early/evolving cellulitis
A score or clinical decision support system	Simple to use Familiar format Provides reassurance/confidence No extra equipment or costs May aid in identifying alternative diagnoses	Perceived as insufficiently specific to rule out inflammatory mimics May be time-consuming if data entry is lengthy Relies on subjective inputs, only as good as the data put in Could promote risk-averse decision-making, feel overly prescriptive Concerns about blame if results are over-ruled
Point-of-care blood test (eg, C reactive protein)	Simple to use Rapid knowledge of inflammatory markers would be useful	Perceived as insufficiently specific to rule out inflammatory mimics C reactive protein (CRP) rises can be delayed Risk of focusing on the number over clinical judgement May detract from holistic patient assessment

#### Emerging/novel technologies

Participants discussed ideas for novel diagnostic tools for cellulitis, as outlined below. Associated quotes are presented in table 4. Novel ideas included a wearable device (like a home blood pressure machine) in the form of a sock/plaster/bandage that might have a built-in thermometer or change colour when bacterial toxins are detected. Concerns existed regarding potential discomfort caused by any device that needs to touch affected skin, whether it would work if blisters and discharge were present and the possibility of cross-infection between patients.

**Table 4:** Opinions regarding emerging/novel technologies

	What could work	Potential issues
Contact-based methods	“with that ageing population we need to have more things done in the community by people to themselves. So something that’s nice and simple, you know, just like people would use their own thermometer or blood pressure machine…like a plaster that you put over the area that, you know maybe got a built in thermometer and you can like detect bacterial toxins or something, you know. Maybe even…something as simple as changing colour” (P21 GP, OOH)	“something that involved sticking electrodes in the skin – or taking skin scrapings…That’s gonna be less preferable to a thermal scanner which they can’t feel, or a plaster that just sort of sticks to the skin.” (P21 Advanced Nurse Practitioner) “If you’ve got blistering, bullous cellulitis or if it’s weeping, would it work, would it stick, would it adhere? Also that ouch [slight laugh), I just think of the pain.” (P6 lead antimicrobial pharmacist)
Imaging technology	“Oh, so some sort of scan thing like a point of care ultrasound type thing or, you know, the vein finders that nurses use in ED. That sort of thing would be ideal. So not messing around with bodily fluids” (P2 GP, OOH) “with cellulitis, more than anything else, there is a visual aspect of it that it’s important to take into account. So, anything that would be able to give an idea of how things have progressed over time ‘cause they might not be the same professionals that see patients every day, in fact, most of the times, they are not.” (P22 infectious diseases and microbiology consultant) "So somebody’s coming to see whether it’s a cellulitis or a DVT.…It’ll be nice to have a tool which does both….the portable ultrasound built-in with infrared scanner.” (P12 microbiology consultant) “virtual technology so that they could offer their services to a lot of areas, a lot of clinics at the same time. So by rotation…they would have somebody who would be able to conduct the diagnostics locally if required through a nice 3D camera, so that they could see the live images at a depth, they could speak to the patient” (P11 infectious diseases consultant)	“I don’t think you’re going to be able to diagnose cellulitis with a scanner, well maybe you could if you had some sort of really fine ultrasound scanner so you could look at the dermis or something like that to see if there’s infection there, but I suspect some combined blood test combined with some sort of algorithm probably” (P18 acute medicine consultant) “I think from a primary care point of view…I don’t think a scan of some sort would be feasible to do like if you’re doing a home visit or even if it’s like a mobile, some sort of a mobile scanning device… first of all, you’d need a lot of training for that. So I think realistically it’s probably more some kind of the on the spot blood test.” (P13 GP, salaried) “But I am aware that true cellulitis involves several layers of the skin…I would imagine that ultrasound would pick up the more severe deeper cellulitis? But would it pick up the cellulitis in the more superficial layers? How easy would it be to interpret because at the end of the day the end user would be the doing the interpretation. But if you paired that with artificial intelligence, then you might be on to a bit of a winner.” (P6 lead antimicrobial pharmacist)
Artificial intelligence-assisted device	“Right so we are living now in the artificial intelligence sphere…so I presume that there is some sort of intelligent device on the horizon that will help doctors get the job done I wouldn’t say in a perfect way but I will say in a better way.” (P14 acute medicine and cardiology consultant) “In my head it’d be really cool if it was almost like something where you could scan the leg and it automatically, I don’t know how it would do that, but something that you could scanning over and it would somehow magically tell you, I don’t know the amount of inflammation and the depth of the inflammation.” (P25 advanced clinical nurse practitioner)	

Participants suggested imaging devices, such as a three-dimensional camera, a scanner to indicate bacterial concentrations within tissues and a portable ultrasound device. Some HCPs did not have confidence that current ultrasound methods would be able to diagnose cellulitis accurately. Pairing ultrasound with artificial intelligence (AI) or using a different AI-assisted device were alternative suggestions. Concerns included the potential complexity of imaging devices and the requirement for a high level of training. Some participants suggested that a patient could use one of the simpler devices at home (potentially as part of a ‘rescue pack’ for those with recurrent infection) and send the result to an HCP.

### Theme 4: characteristics of an ideal cellulitis diagnostic test/tool

Here, we present HCPs’ perceptions of the ideal characteristics of a diagnostic test. While many features would be useful across a range of different use cases, table 2 links specific ideal test characteristics to use cases identified by participants.

#### Settings

Most participants suggested the test would be useful in community and hospital settings whenever the point of first contact was in the patient’s pathway. However, some also suggested that a test could be used to revisit a preexisting diagnosis (‘previous oral antibiotic therapy’ and ‘challenging a premade diagnosis’ subthemes above), which would happen after the first point of contact.

HCPs were asked how frequently they anticipated using a diagnostic test for cellulitis in their clinical setting, and their answers were typically expressed as the number of times per day or per week. The anticipated frequency of use was the highest in emergency departments, followed by urgent treatment centres, the acute medical take, primary care and other community settings (eg, home visits by district nursing teams, long-term care facilities) and the lowest in dermatology clinics.

#### Users

Participants described a range of potential users, essentially any clinician querying cellulitis, including community nurses, emergency nurse practitioners, paramedics, healthcare assistants, GPs, other doctors and pharmacy prescribers. Some HCPs stated that patients and carers could be the users. Others reported that a diagnostic test or tool would be a beneficial aid for doctors with less clinical experience and HCPs who are not doctors, because the more times you have encountered the condition the easier it is to differentiate from mimics.

“I mean it might be if it’s like a finger prick test, then nursing home staff can…relay the results to GP and out of hour GP so that might be useful.” (P13 GP, salaried)

“So I am a big fan of giving patients the power and as early in the pathway as possible. So in my ideal world, there’ll be something that the patient could administer at home and then make the decision themselves as to whether they needed to say call 111, see their GP or whether they could self-manage.” (P2 GP, OOH)

#### Time to result

Most HCPs’ preferred time to result was very short (<15 min), reflecting the time constraints on appointment length and how long the current diagnostic process takes. A few HCPs working in secondary care would accept longer wait times of up to 1 hour but would prefer shorter ones and only one HCP suggested that longer than this would be acceptable.

“so imagining you’re on a home visit, which, you know, very limited time in your lunch hour and, or a community nurse who’s got to be somewhere next, so I’m guessing, you know, the longest could be probably ten minutes.” (P1 GP, salaried)

#### Result format

Most participants felt that nothing in medicine has a clear yes/no answer and preferred to avoid this format. Example formats included a percentage, low/medium/high or traffic light system of the likelihood of a cellulitis diagnosis. HCPs also said that, alongside the result, an interpretation or an action point could be helpful, for example, high probability, antibiotic prescription recommended.

“That’s why I think the clinical risk scores are quite nice because then you can use that alongside a scoring system and you can back up your decision making and it’s an aid rather than a yes or no.” (P8 acute medicine consultant)

However, a more definitive answer was considered to be beneficial for certain users, particularly if they were relaying the information to a prescriber.

“So what I would like is for it to give a clear, unambiguous thing. So not like an ECG, where you need expertise to use it. I mean, in my ideal world, I’d like something I can send out a healthcare assistant with and it would tell me, yes.” (P2 GP, OOH)

There was a division of opinion on whether a panel of tests covering some key differential diagnoses would be helpful.

“I don’t think there’s going to be a single test that’s going to allow you to distinguish between cellulitis and another diagnosis, I think a panel of tests combined with a risk score is probably going to be your best bet to give you your either black or white or percentage chance more likely of whether it’s cellulitis or not.” (P18 acute medicine consultant)

#### Training requirements

HCPs reported that the time allocated to training should be 30 minutes or less, but durations between 1 hour and 4 hours were also mentioned. The duration would depend on the type of test, its usefulness and the time existing clinical commitments and employers would realistically allow. The type of test would also impact the preferred format of training, whether online or combined face-to-face and online training.

“We tried online stuff with things like dopplers and things like that and people just aren’t confident after them. They really prefer a hands-on approach where they can actually speak face to face with a trainer and get hold of the device and use it on each other.” (P25 advanced clinical practitioner)

HCPs also offered other ideas to help with adoption which included prompts that would be useful to have post-training. For instance, a voice module that talks you through the steps (like the set-up process on some smartphones) or a six-step poster guide. A departmental staff member who could serve as a trainer (and provide troubleshooting) was also felt to be useful.

“Then it’s really useful to have somebody almost as the champion within the team who can train everybody else up on the one that is in the vicinity, that seems to work quite well.” (P5 advanced district nurse specialist)

#### Performance

Most HCPs reported that a highly sensitive test is more important than a highly specific one because the consequences of missing the diagnosis are viewed as more harmful than treating cellulitis incorrectly. Overall, 56% (14/25) participants prioritised sensitivity, 36% (9/25) prioritised specificity and 8% (2/25) did not express a clear preference.

“because actually the costs and the implications of overtreating is…coming for a couple of days with some antibiotics and we’re not going to have those antibiotics in the future as much. However, undertreating them means a hospital admission, they’re potentially going to be septic…all the cost implications of that, core patient experience, lack of trust in healthcare professionals, so they’ll end up coming back quicker, if that happens again, and they’ll get misdiagnosed more. (P17 emergency nurse practitioner)

18 participants considered 90% as an acceptable sensitivity level for a test. Reasons included that no test would ever achieve 100% accuracy and that the test would be used alongside clinical judgement. Four participants felt that missing 1 in ten cases was unacceptable, largely because clinicians might avoid using a test perceived to miss too many cases and prescribe antibiotics regardless. One participant considered 85% sensitivity acceptable while two did not provide a clear threshold because they felt acceptable sensitivity would depend on factors such as where the test was used in the clinical pathway and whether it was administered by HCPs or patients.

A smaller number of participants, mainly those more involved in antimicrobial stewardship (eg, antimicrobial pharmacists, microbiology consultants), regarded the test’s specificity as more important.

“So I’d rather it miss someone with cellulitis I think than over diagnose, because I think clinicians would always have the tendency to trust their gut and go with something if a test is telling them it’s not this and they’ll go, well, it is this” (P10 consultant antimicrobial pharmacist)

Views regarding specificity were more variable. 10 participants considered a false-positive rate of 20% acceptable. Nine participants felt that 20% was too high but did not specify an alternative threshold. Two participants suggested acceptable false-positive rates of 10% and 10–15% respectively. Four participants did not provide a clear threshold because they felt the question depended on the consequences of incorrect labelling or was too difficult to answer.

The current standard of care was used by some participants as a benchmark for acceptable performance (participants were informed that approximately one-third of cases are misdiagnosed, although the most recent estimates from the literature are now closer to 40%).^4^

“Well, I guess I think you have to compare it to a third. You know, that statistic. You know, if it kind of can't surpass that level, then there’s no point really…is there?” (P4 GP, locum)

### Practicalities

Most participants felt that annual calibration was acceptable. Weekly or monthly calibration was undesirable and after every use or daily was unacceptable to most. Some participants highlighted the need to clarify decontamination procedures. In terms of power requirements, HCPs suggested that devices be rechargeable within a good timeframe to enable portability between and around settings. For troubleshooting assistance, participants felt that either an online quick guide with frequently asked questions or a callback/email response within 24–48 hours would be adequate.

## Discussion

### Key findings

In this qualitative interview study of UK HCPs, we found that cellulitis remains a challenging diagnosis, particularly in patients with atypical or recurrent presentations, pre-existing wounds, multiple comorbidities, darker skin tones or prior antibiotic treatment. Participants highlighted that the ease of diagnosing and managing cellulitis, combined with lack of knowledge and limited access to dermatology or tissue-viability expertise, contributed to overdiagnosis. Antibiotics were frequently prescribed because clinicians viewed missing infection as riskier than overtreatment and patient expectation for antibiotics or concern about contradicting another HCP reinforced prescribing.

Some benefits of candidate diagnostic aids such as thermal imaging, scoring systems and point-of-care tests were recognised, but participants doubted their specificity. Participants were interested in novel technologies but cautious about infection control issues, discomfort and training needs. A few felt AI might support interpretation. An ideal test was described as rapid, portable and usable by many HCPs, providing a probability rather than binary result.

### Strengths and limitations

Strengths include that we captured views on candidate diagnostic aids, emerging novel tools and desired test characteristics, information that is vital for device design. Another strength is the breadth of perspectives across generalist and specialist roles as, compared with earlier qualitative work, our sample contained fewer dermatology specialists.

Our findings highlight that different professional groups may have different perspectives on the role of a diagnostic test, with most participants prioritising sensitivity to avoid missing true cellulitis, while those working in antimicrobial stewardship placed greater emphasis on specificity to minimise unnecessary antibiotic use. Nevertheless, diagnostic accuracy is a complex concept and would merit further detailed discussion to establish acceptable target performance.

Limitations include that the hypothetical framing of some questions (eg, training requirements for an unspecified device) may have limited the relevance of responses. When exploring sensitivity and specificity with respondents, we ensured understanding of these concepts by using example thresholds of 90% sensitivity and 80% specificity (see online supplementatry materials, interview topic guide), which may have influenced participants’ views regarding acceptable thresholds. The sensitivity and specificity of certain diagnostic tests (eg, thermal imaging) have been reported in the literature. However, due to time constraints and the breadth of topics covered, we did not present these data during interviews and therefore do not know whether this knowledge may have altered participants’ views on candidate diagnostic aids. Specifically for thermal imaging, the most recently reported sensitivity and specificity estimates using maximum skin temperature difference are 93.5% and 38.4%, respectively, which would likely have been acceptable to most participants with regard to sensitivity, but not specificity.^14^ While we explored acceptable test performance, we did not investigate what probability threshold (eg, percent likelihood of cellulitis) clinicians would require before diagnosing and managing an individual patient as cellulitis. Finally, as a UK-based study, findings may not fully transfer to health systems with different patient pathways, primary and secondary care interfaces or specialist access.

### Findings in the context of other evidence

Patel and colleagues interviewed 20 UK HCPs and found diagnostic confidence increased with experience.^15^ Similarly, several HCPs in our study thought less-experienced clinicians would benefit most from diagnostic support. Both studies highlighted dermatology expertise as key to recognising mimics. Cellulitis has been described as ‘a disease with no home’,^16^ and it is perhaps this wide distribution across multiple specialties that contributes not only to misdiagnosis but also to patient perceptions of receipt of insufficient information on treatment and prevention.^17^

Patel *et al* also identified recurrent disease, chronic skin changes and darker skin as diagnostic challenges. However, a new issue repeatedly raised by our participants was the assessment of patients with pre-existing wounds or ulcers, which they consider along with cellulitis and treat with antibiotics when thought to be infected. The challenge arises when it is uncertain whether infection is truly present or whether the wound or ulcer is simply poorly managed, reflecting limited skills and restricted access to tissue-viability services. Another novel finding from our study was the tendency toward diagnosing cellulitis when uncertain because it was ‘the default diagnosis’, reflecting the relative ease of treatment and follow-up compared with mimics.

Pulia and colleagues interviewed clinicians in US emergency departments to explore barriers to optimal antibiotic use.^18^ Like Patel *et al* and our study, they found fear of adverse outcomes drove overtreatment, but uniquely described that there was an organisational culture where missing infection was unacceptable. Pulia *et al* also added that there was a system (hospital/departmental) pressure to ‘do something’, a factor not reported in our UK sample and reflecting the differing healthcare systems. Similar to our findings, they also reported the challenge of patient expectations for antibiotics, although other research suggests that some clinicians may overestimate patient expectations, which are more nuanced and can be influenced by the HCP behaviour.^19^ Clinicians in their study also reported they were more likely to prescribe antibiotics for higher-risk patients.

Griffin *et al* used a case vignette survey to emergency department clinicians and found that combining diagnostic tools (eg, thermal imaging) with patient education and shared decision-making, rather than using them in isolation, resulted in the lowest antibiotic prescribing probabilities.^20^

### Implications for practice, policy and research

Improving cellulitis diagnosis requires better recognition of common non-infectious mimics such as venous stasis or eczematous dermatitis and oedema/lymphoedema.^4^ These conditions lack more objective diagnostic tests, unlike deep vein thromboses or gout, and clearer pathways for their recognition and management would help reduce reliance on cellulitis as the default diagnosis. As many HCPs regard cellulitis and infected pre-existing wounds or ulcers as a single clinical entity, distinguishing which patients require antibiotic therapy and adjunctive measures (eg, dressings, tissue care) from those with non-infective inflammation would be particularly valuable. Expanding rapid access dermatology and tissue-viability services could reduce misdiagnosis but may be costly.

Although most HCPs in our study prioritised sensitivity in diagnostic performance, experiences of misdiagnosis largely reflected over-calling cellulitis rather than missing it, and negative views of all candidate diagnostic aids were that they were insufficiently specific. Recent prospective emergency department data from Pulia *et al* suggested that thermal imaging and a clinical scoring system could theoretically reduce cellulitis overdiagnosis by 8.9% and 5.7%, respectively, while combining both approaches reduced overdiagnosis by 13.0%.^21^ Recent trials on cellulitis treatment have explored whether antibiotic duration can be shortened safely.^22 23^ However, a highly specific test that could rule out mimics and reduce the estimated 40% of misdiagnosed cases would yield a far greater impact on antimicrobial stewardship by avoiding treatment entirely. It was HCPs most involved in antimicrobial stewardship who preferred higher specificity.

Future diagnostic tools should be developed to require minimal training, produce results ideally within minutes and function in both community and hospital settings. Developers must ensure comfort, infection-control safety and validation across skin tones. Integration with follow-up mechanisms, such as remote image monitoring, could reduce fear of missed infection and enhance safety-netting. Broad stakeholder engagement including patients, carers, generalist clinicians, engineers and commissioners should define acceptable parameters to inform preferred product characteristics for industry.

## Supplementary material

10.1136/bmjopen-2026-117201 online supplemental file 1

10.1136/bmjopen-2026-117201 online supplemental file 2

## Data Availability

No data are available.
