# Supplementary material for: Unmet needs in cellulitis diagnosis: a qualitative interview study to understand healthcare professionals’ knowledge and experiences
Source: BMJ Open. 2026 Jun 28;16(6):e117201. doi: 10.1136/bmjopen-2026-117201 (PMC13311749; doi:10.1136/bmjopen-2026-117201)
Supplement: online supplemental file 1 [file bmjopen-16-6-s001.docx]

I**nterview Topic Guide**

*Participants will be asked to reflect on some cases of cellulitis that they have been involved in the diagnosis and/or management of prior to the interview.*

**Introduction**

The first part of the interview will be about the role that diagnostic technologies or other aids may play in improving the diagnosis and management of cellulitis.

Then we will move onto the second part, which will focus on what you would consider to be the key aspects of an ideal diagnostic to differentiate cellulitis from other conditions with similar clinical presentations.

**Part 1**

We are now going to discuss if diagnostic technologies or aids could help in improving the diagnosis and/or management of cellulitis. It would be useful to do this by considering some cases of cellulitis where you may have found the diagnosis or management particularly challenging.

- What challenges have you come across in the diagnosis of cellulitis?
- What are the key differential diagnoses of cellulitis that you usually consider?
- What challenges have you come across in the management of cellulitis?
- Where in the patient’s clinical pathway do you think a technology or aid would have been useful? [see prompts]
- Do you have any ideas about what the technology would have looked like or how it would work?
- How would it have been used in the decision-making process?
- How would the result have helped you?
- How would the result have helped the patient?

**Prompts:**

Can you think of any diagnostic technology or aid that may help to determine whether:

- to admit / refer for admission patients with cellulitis?
- to treat with intravenous or oral antibiotics?
- to switch from intravenous to oral antibiotics for cellulitis?
- a patient is responding to antibiotics for cellulitis and when you should stop antibiotics?

**Part 2**

We are now going to explore what you perceive to be the key aspects of an ideal diagnostic test or aid which could be used to differentiate cellulitis from other conditions with a similar presentation.

- If participant has not already discussed the key differential diagnoses – what are the key differential diagnoses of cellulitis that you usually consider?

Prompts: DVT, thrombophlebitis, venous eczema, lymphoedema, chronic heart failure causing oedema, allergic reaction to insect bites, lipodermatosclerosis, erythema nodosum, necrotising fasciitis. Over joint: Gout, septic arthritis, bursitis

- How would it help to have a diagnostic technology or aid to prevent the misdiagnosis of cellulitis (note ~1/3 of patients are misdiagnosed as having cellulitis)
- Thinking about the clinical pathway of patients with cellulitis, in what setting do you think a diagnostic test would be most useful?

*Test Application/functionality*

- What different environments do you think the test needs to be able to work in?
- Which healthcare professionals do you think should have access to the test?
- Which healthcare professionals do you think should be able to operate the test?
- What is the maximum amount of time that training to use the test should take?
- What would be your preference for the format of training (e.g. interactive online tutorial, online video, face to face)?
- What would be the maximum amount of time taken to do the test (or steps required) that would be acceptable to you? Why this amount? Is this applicable to both clinicians and patients?
- What power requirements for the test would be acceptable (e.g. no power required, batteries, mains electricity)?
- What maintenance requirements for the test would be acceptable?
- What level of support or troubleshooting assistance for the test would be acceptable?

*Test Result Output*

- How quickly do you think you would need the answer for it to improve management/be useful?
- What format would it be useful for the results be in? Given there is a differential diagnosis, would it be solely a result to determine if the diagnosis if cellulitis or would a panel of tests be useful (e.g., inflammatory markers + d-dimer + uric acid)?
- Would the results be something that could be shared with patients?
- What level of detail do you think you need to know? What is the causative microorganism? What antibiotics is the bacteria resistant to?

*Test Performance*

- If you had 100 people with cellulitis and the test only correctly identified 90 of these people, would that be an acceptable level of performance? If yes/no, why? How many people of 100 would it have to correctly identify to be acceptable?
- Often tests are a trade off, and if the test is very good at identifying cellulitis, then it may also be more likely to incorrectly label people as having cellulitis who do not. Would it be acceptable if 20 out of 100 patients without cellulitis were labelled as having cellulitis and received unnecessary antibiotic treatment? If yes/no, why? How many of 100 healthy people would be the maximum the test could label incorrectly as having cellulitis?
- Which of these measures of performance do you think is more important?
- If this diagnostic test was able to accurately differentiate a case of cellulitis from another cause, what expenditure would this avoid (e.g., cost of antibiotics, cost of ambulance transfer, cost of ambulatory care attendance)? How much do you think the test could save?
- Are there any other features that you think would be important?
- Are you aware of any newly developed diagnostic tests which could differentiate cellulitis from other conditions with a similar presentation?

**Prompts:** If not discussed already or if participants are struggling to answer any of the questions, then can discuss that there has been a small number of observational studies conducted on potential new diagnostics and what their views on these are:

- Thermal imaging
- Point-of-care tests
- Clinical Prediction Models
- Computerised diagnostic decision support systems
